# Reactive Oxygen Species (ROS), Intimal Thickening, and Subclinical Atherosclerotic Disease

**DOI:** 10.3389/fcvm.2019.00089

**Published:** 2019-08-02

**Authors:** Denise Burtenshaw, Michael Kitching, Eileen M. Redmond, Ian L. Megson, Paul A. Cahill

**Affiliations:** ^1^Vascular Biology & Therapeutics, School of Biotechnology, Dublin City University, Dublin, Ireland; ^2^School of Chemistry, Dublin City University, Dublin, Ireland; ^3^Department of Surgery, University of Rochester, Rochester, NY, United States; ^4^Centre for Health Science, UHI Institute of Health Research and Innovation, Inverness, United Kingdom

**Keywords:** NOX, NAPDH oxidase, smooth muscle (physiology), endothelial cells, adventitial cells, stem cells, intimal thickening, arteriosclerosis

## Abstract

Arteriosclerosis causes significant morbidity and mortality worldwide. Central to this process is the development of subclinical non-atherosclerotic intimal lesions before the appearance of pathologic intimal thickening and advanced atherosclerotic plaques. Intimal thickening is associated with several risk factors, including oxidative stress due to reactive oxygen species (ROS), inflammatory cytokines and lipid. The main ROS producing systems *in-vivo* are reduced nicotinamide dinucleotide phosphate (NADPH) oxidase (NOX). ROS effects are context specific. Exogenous ROS induces apoptosis and senescence, whereas intracellular ROS promotes stem cell differentiation, proliferation, and migration. Lineage tracing studies using murine models of subclinical atherosclerosis have revealed the contributory role of medial smooth muscle cells (SMCs), resident vascular stem cells, circulating bone-marrow progenitors and endothelial cells that undergo endothelial-mesenchymal-transition (EndMT). This review will address the putative physiological and patho-physiological roles of ROS in controlling vascular cell fate and ROS contribution to vascular regeneration and disease progression.

## Introduction

Arteriosclerosis occurs when the arterial blood vessels that carry oxygen and nutrients from the heart to the rest of the body become thick and stiffen thereby restricting blood flow to vital organs (1). It is a common feature of aging while pulmonary hypertension, peripheral arterial disease (PAD), transplant arteriosclerosis and in-stent restenosis (ISR) following balloon angioplasty are all significant clinical outcomes peroxidation (2–5). Atherosclerosis is a specific type of arteriosclerosis and refers to the specific build-up of lipids, cholesterol and other substances in and on the artery wall forming a plaque which can further restrict blood flow (6). It is considered the main cause of cardiovascular disease and is characterized by the early development of subclinical atherosclerosis due to pathologic intimal thickening (PIT) within atherosclerotic-prone regions of the vasculature (7). Subclinical atherosclerosis is an early indicator of atherosclerotic burden and its reversal can prevent the progression to symptomatic cardiovascular disease (CVD) (1). For the purpose of this review, subclinical atherosclerosis refers to the early intimal thickening that occurs prior to the accumulation of lipid and early plaque formation (8).

Reactive oxygen species (ROS) are a class of highly reactive molecules derived from O_2_ metabolism (9). Members of the ROS family include: superoxide (O_2_−), alkoxyl radical (RO·), peroxyl radical (ROO·), hydroxyl radicals (OH·), peroxynitrate (ONOO−), hydrogen peroxide (H_2_O_2_), ozone (O_3_), and hypochlorus acid (HOCl). Physiological concentrations of ROS are important signaling molecules that maintain vascular homeostasis whereas excessive ROS production may result in oxidative stress leading to vascular disease progression. ROS maintain vascular cell homoeostasis by controlling the phenotype and fate of multiple cell types including endothelial cells (ECs), vascular smooth muscle cells (SMCs), adventitial cells, myeloid cells and resident stem/progenitor cells (10, 11).

Oxidative stress influences the onset and progression of subclinical atherosclerosis by inducing early endothelial cell activation, permeability changes to the endothelium, disruption of glycocalyx, activation of myeloid and progenitor stem cells leading to the eventual accumulation of vascular smooth muscle (SMCs)-like cells within the intima (12–14). This accumulation of cells promotes diffuse (DIT) and adaptive (AIT) intimal thickening and is considered an important nexus in the development of subclinical atherosclerosis. These cells may originate from (i) medial SMCs (15), (ii) resident vascular stem cells (16) (iii) circulating bone marrow-derived mesenchymal stem cells (17) and (iv) endothelial cells undergoing mesenchymal-stem-cell-transition (EndMT) (18). Concurrently, innate and adaptive immune cells that enter the vasculature may also participate in the pathology of hypertensive-induced arteriosclerosis by releasing several mediators including ROS that cause vascular damage leading to adaptive (AIT) and pathologic (PIT) intimal thickening (19). In this context, ROS affect resident myeloid cells that reside within intimal and/or adventitial layers of susceptible regions of arterial vessels that display low-grade inflammation (20–22). Upon exposure to hypercholesterolemia, these cells become laden with oxidized LDL and acquire foam cell morphology prior to the recruitment of monocytes that differentiate into macrophage foam cells (6).

**Figure 1:**
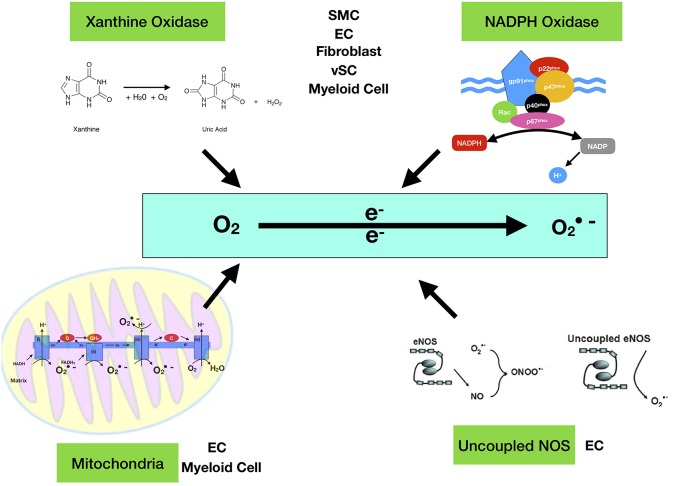
Enzymatic sources of superoxide anion (·O_2_−). The major enzymes responsible for ROS generation in the vasculature include mitochondria (mtROS), NAD(P)H oxidase, xanthine oxidase, and uncoupled NOS. NAD(P)H oxidase is a multi-subunit enzyme, comprising gp91phox (or its homologs, NOX1 and NOX4), p22phox, p47phox (or NOXO1), p67phox (or NOXA1), and p40phox. Smooth muscle cell (SMC), endothelial cell (EC), Myeloid Cell (monocytes and macrophages), vSC (vascular stem cell). The mitochondrial electron transport chain produces mtROS. Mitochondrial complexes I and II use electrons donated from NADH and FADH2 to reduce coenzyme Q during the process of oxidative phosphorylation (OXPHOS). Leakage of electrons at complex I and complex III from electron transport chains leads to partial reduction of oxygen to form superoxide [Quinol QH_2_, quinone Q and C cytochrome c].

Once thought to be nothing more than harmful by-products of cellular metabolism, it is now clear that low—moderate ROS levels contribute to cellular functions such as differentiation, migration, adhesion, senescence, growth and apoptosis (23). The main ROS producing systems *in-vivo* are reduced nicotinamide dinucleotide phosphate (NADPH) oxidase (NOX) (11), xanthine oxidase (XO) (24), the electron transport chain in the mitochondria (25), cytochrome P450 (26), lipoxygenases, heme oxygenase and cyclooxygenases (27), myeloperoxidase (28), monoamine oxidases (29) and uncoupled nitric oxide (NO) synthase (30). ROS can also be generated from exogenous sources such as UV light, air and water pollution, alcohol, tobacco smoke, transition and heavy metals, industrial solvents, pesticides, high temperature (31) (Figure 1). Table 1 lists the seven isoforms of NOX expressed in mammals. While, NOX represents the major source of vascular superoxide anion that generates oxidative stress (45), endothelial ROS is also generated in the mitochondria from the partial oxygen reduction to form superoxide and also participates in the activation of these cells following cholesterol loading (46). Similarly, macrophages produce elevated levels of mitochondrial ROS in a NOX-independent fashion (47).

**Table 1:** Isoforms of NOX.

**Isoform**	**Expressed**	**Role**	**References**
NOX1	Colon, vascular smooth muscle cells, and stomach	Cell growth	(32, 33)
NOX2	Endothelial cells and neurons	Phagocytosis. Greatly implicated in disease states due to association with inflammation.	(34, 35)
NOX3	Mainly in fetal kidney and liver tissue. Also in inner ear.	Development of otoconia in the inner ear	(36–38)
NOX4	A variety of cell types including vascular endothelial, smooth muscle and mesenchymal stem cells	DNA damage	(39–42)
NOX5	Lymphocytes of spleen and lymph nodes and in testes	Calcium dependent enzyme	(43)
DOUX1	Thyroid	Thyroid hormone synthesis and host defense	(44)
DOUX2	Thyroid	Thyroid hormone synthesis and host defense	(44)

Initially O_2_− is formed from the reduction of molecular O_2_. O_2_− is the most pathologically relevant molecule due to its high chemical reactivity, therefore O_2_− requires rapid reduction to H_2_O_2_ by the enzyme superoxide dismutase (SOD) (48). H_2_O_2_ is thought to be the main ROS molecule involved in intracellular signaling. This reaction may also occur spontaneously in a process known as dismutation. O_2_− may react with H_2_O_2_ in the presence of iron (released from O_2_− oxidative damage to proteins containing FeS clusters) to generate damaging OH- radicals, or O_2_− may also react with NO to form ONOO− (31). ROS are important mediators and signal modifiers upon stimulation by growth factors (49, 50), cytokines (51), hypoxia (52), shear stress (53), and cyclic strain (54). In response, many important pathways are activated such as GPCR, Notch, Wnt-β-catenin, MAPK, JAK-STAT, NF-κB, and PI3K/AKT (55–60).

ROS acts as an intracellular signal through reversible oxidation of amino acid residues, most commonly cysteine (61). This induces a conformational change in the sensor protein and influences their function, stability, subcellular localization and protein-protein interaction. H_2_O_2_ is the most studied ROS mediator due to its stability and its ability to diffuse through the phospholipid bi-layer (62). H_2_O_2_ has been implicated in a variety of cellular processes (63), including proliferation and migration (64), differentiation (65), and apoptosis (66). O_2_− signaling is less understood due to its poor stability and the difficulty to specifically target O_2_− *in vitro* or *in vivo* (67). In spite of its low stability and poor diffusion, it can oxidize thiol groups of proteins in the immediate vicinity of where it was generated (68). O_2_− signaling has been associated with major epigenetic processes, including DNA methylation, histone methylation and histone acetylation (69). ROS also possess antimicrobial functions, important in phagocytosis and pathogen destruction (70).

Generation of ROS is tightly regulated by the ROS scavenging system, which are enzymes that neutralize ROS. These include SOD, catalase, heme-oxygenase-1 (HO-1), NADPH quinone reductase and, gamma-glutamylcysteine reductase (48). Oxidative stress is normally induced when the production of ROS overcomes the ROS scavenging system. This facilitates lipoprotein/phospholipid oxidation, protein denaturation, and DNA damage through free-radical-mediated chain reaction, primarily through the reduction of guanine residues to 8-oxoguanine (71). OH· radicals can also cause single/double strand breaks in DNA (71). The anti-oxidant defense response, primarily SOD, regulates ROS signaling by limiting the concentration of ROS to low or moderate levels, controlling the redox profile of the cell and ensure that ROS are localized close to their intended targets (70). SOD1 inhibition by tetrathiomolybdate increased intracellular O_2_− and H_2_O_2_ levels and attenuated growth factor mediated ERK1/2 signaling in endothelial and tumor cells (48). Glutathione peroxidase (GPx-1) has also an important anti-oxidant role in the generation of ROS. GPx-1 is inversely associated with CVD and important for maintenance of a normal level of GSH. It can also protect mitochondria against ROS-induced reoxygenation damage *in vivo* (72).

The overall consensus is that ROS production when not compensated for by scavenging endogenous antioxidants will lead to the rise of ROS beyond a “normal” or “physiological” threshold level. This results in a process termed “oxidative stress.” Intracellular ROS generation may be pathological or physiological (73). ROS is invariably generated from cellular metabolism or in response to various exogenous stimuli. While the main endogenous source of ROS is the electron transport chain of the mitochondria and cytosolic generation by NOX, other ROS sources are referred to as “professional” generators, capable of producing high levels of ROS in a spatial and temporal manner (74). NOX derived ROS has been implicated in cancer (75), diabetes (76), neurodegenerative disorders (77) and CVD (78).

## Vascular Mitochondrial ROS (mtROS)

Mitochondria are unique in that they are not only a major source of ROS but are also particularly susceptible to oxidative damage by ROS. Consequently, mitochondria suffer oxidative damage with age that contributes to mitochondrial dysfunction (79). Under physiological conditions, mitochondrial metabolism results in the build-up of potentially damaging ROS which are neutralized by mitochondrial permeability transition pore (mPTP) openings that maintain healthy mitochondrial homeostasis. However, adaptive and maladaptive responses can occur that involve activation of mitochondrial channels such as mPTP and inner membrane anion channel (IMAC) resulting in intra- and intramitochondrial redox-environment changes leading to ROS release. Physiological levels of ROS produced in the mitochondria (mtROS) are critical components of downstream signaling pathways including those regulating immune responses and autophagy (59, 80).

Mitochondria have a four-layer structure, including outer mitochondrial membrane, intermembrane space, inner mitochondrial membrane, and matrix. Generation of mtROS occurs during the process of oxidative phosphorylation (OXPHOS) at the electron transport chain (ETC) located on the inner mitochondrial membrane. Five big protein complexes are involved in this process. These ETC complexes are named complex I (NADH dehydrogenase (ubiquinone), 45 protein subunits), complex II (succinate dehydrogenase, 4 protein subunits), complex III (ubiquinol-cytochrome c reductase, 10 protein subunits), complex IV (cytochrome c oxidase, 19 protein subunits), and complex V (ATP synthase, 19 protein subunits). Electrons donated from nicotine adenine dinucleotide (NADH) at complex I and flavin adenine dinucleotide (FADH2) at complex II pass through ETC and ultimately reduce O_2_ to water at complex IV. Positively charged protons (H^+^) are actively pumped from the mitochondrial matrix into the intermembrane space, resulting in the increased negative charges in the mitochondrial matrix and the upregulated positive charges in the intermembrane space, and thus creating a mitochondrial membrane potential (Δψm) across the inner mitochondrial membrane. This proton-motive force allows complex V - ATP synthase (ATP-ase) to generate ATP from adenosine diphosphate (ADP) and inorganic phosphate when protons re-enter the mitochondrial matrix through the complex V enzyme. However, the process of ETC is not perfect and leakage of electrons occurs at complex I and complex III resulting in partial reduction of oxygen to form superoxide (O_2_.−). It has been estimated that 0.2–2.0% of O_2_ consumed by mitochondria generates the superoxide (O_2_.−). There are three leakage events: complex I leaks O_2_.− toward the mitochondrial matrix, while complex III leaks O_2_.− toward both the intermembrane space and mitochondrial matrix (79) (Figure 1).

Overall, there are 11 sites of ROS production (superoxide and/or hydrogen peroxide) identified in mammalian mitochondria related to substrate metabolism, electron transport and oxidative phosphorylation (81). However, because mtROS and ATP production are both coupled to electron transport chain activity, it is unclear how mtROS is induced independently of ATP synthesis. Recent studies now suggest that mtROS is activated via unique calcium entry–mediated increase of proton leak and mitochondrial O_2_ reduction (46).

The regenerative cycle of mtROS formation and release is termed ROS-induced ROS release (RIRR). Reversible mPTP channel opening and associated ROS release constitutes an adaptive housekeeping function of potentially toxic levels of ROS (and Ca2^+^). At higher ROS levels, longer mPTP channel openings release a ROS burst leading to destruction of mitochondria, and if propagated from mitochondrion to mitochondrion, of the cell itself. The destructive function of RIRR serves a physiological role through removal of unwanted cells or damaged mitochondria. It may however also cause the pathological elimination of vital and essential mitochondria and cells. The adaptive release of sufficient ROS into the vicinity of mitochondria may also activate local pools of redox-sensitive enzymes involved in protective signaling pathways that limit ischemic damage to mitochondria and cells in that area. Maladaptive mPTP- or IMAC-related RIRR may also be playing a role in aging (79).

Collectively, both O_2_.− and H_2_O_2_ are considered the primary mtROS but have different fates. Given its electrophilic property and short half-life, O_2_.− undergoes radical-radical reaction with nitric oxide (NO) to form peroxynitrite (ONOO2.−) within mitochondria, a detrimental oxidant capable of induction of DNA damage, disruption of mitochondrial integrity, and irreversible modification of protein. In contrast, H_2_O_2_ is electrophobic and more stable and hence abundant within mitochondrion a (>100 times greater than that of O_2_.−) thereby rendering mitochondrial H_2_O_2_ an ideal signaling molecule in mammalian cells (63).

Importantly, ROS generation in the mitochondria appears to be an important aspect of ROS production for both endothelial cells (82) and intimal myeloid cells (primarily monocytes and macrophages) in atherosclerosis (83, 84).

## Vascular NADPH Oxidase (NOX)

**Figure 2:**
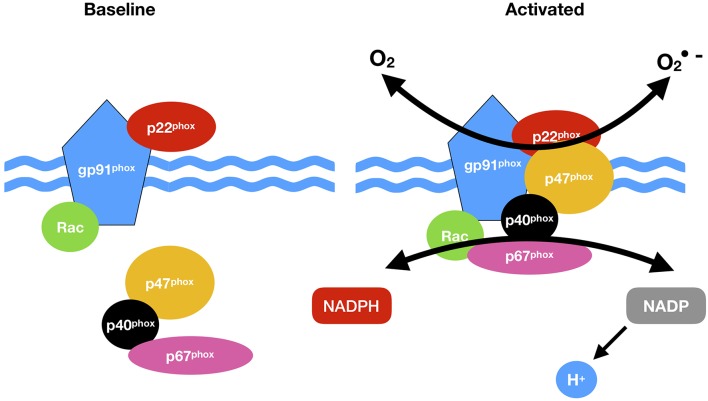
NADPH oxidase (NOX) activation. NOX comprises cytosolic (p47phox, p67 phox, p40 phox, and Rac) and membrane subunits (gp91 phox and p22 phox). During activation of NOX, cytosolic subunits comprise a multi-component enzyme and mi- grate to the plasma membrane to dock with the membrane subunits. This multi-subunit enzyme produces a superoxide anion (O_2_·).

Vascular NADPH oxidases (NOXs) are ROS generating oxidases (85) Table 2. With the exception of NOX5, all NOX enzymes are heteroprotein transmembrane complexes with a core catalytic subunit and a number of regulatory subunits (NOX1, NOX2, NOX4, and NOX5 are expressed and functionally active in human vascular cells. In humans, NADPH oxidase had been thought to be a phagocyte specific enzyme (its catalytic unit: gp91phox) mediating bacterial killing by producing a burst of O_2_− (86). The p22phox, a membrane protein, forms a heterodimer with gp91phox, thereby stabilizing gp91phox and enhancing its O_2_−-producing activity. The ubiquitous expression of p22phox in non-phagocytic cells led to the identification of NOX1, a homolog of gp91phox, in non-phagocytic cells and facilitated the discovery other NOX proteins (45) (Figure 2).

**Table 2:** Vascular NOX isoforms.

**Cell**	**NOX expressed**	**References**
Endothelial cells	NOX1, NOX2, NOX4, and NOX5	(86)
Smooth muscle cells	NOX1, NOX2, NOX4, and NOX5	(87)
Resident MSC-like stem cells	NOX1, NOX2, and NOX4	(88)
Adventitial fibroblasts	NOX1, NOX2, NOX4, and NOX5	(89)
Intimal myeloid cells	NOX1, NOX2, NOX4, and NOX5	(90)

NOX1, 2, 3, 4, and 5 along with Duox1 and 2 and are present in the plasma membrane, endoplasmic reticulum (NOX 2, 4, and 5), the mitochondria membrane and nuclear membrane (87). The classical NOX complex (NOX2) is comprised of the gp91-phox which is the main catalytic subunit that transfers NADPH electrons via FAD and the haem groups to O_2_ and constitutively forms a heterodimer with p22-phox on the membrane (91). Classical NOX is also comprised of three cytosolic subunits p47-phox, p67-phox and p40-phox along with the G-protein Rac (92) (Figure 2).

All NOX homologs have 6–7 transmembrane domains with two haem binding regions containing histidine residues and a NADPH binding region on the intracellular C-terminus to facilitate O_2_− production. The different isoforms of NOX contain homologs of the NOX2 gp91-phox subunit. Structural homology of the catalytic core is preserved within NOX1, NOX3, NOX4, NOX5, DUOX1, and DUOX2, however regulation, localization and function slightly vary across isoforms (93).

NOX1, 2, 3, and 5 mainly produce O_2_−, while NOX4, DUOX1, and DUOX2 generate mainly H_2_O_2._ NOX generates O_2_− by a complex reaction once NADPH binds to the cytosolic COOH terminus. Initially the electrons donated from NADPH are used to reduce FAAD to FADH. FADH is then used to reduce O_2_ on the other side of the membrane (93).

NOX is activated by phosphorylation though phagocytic particles, physiological or pathological cues such as hyperglycaemia, altered cellular hypoxia, and inflammation (76, 94, 95) Phosphorylation of p47-phox may be mediated by several serine kinases including protein kinase C isoforms, mitogen-activated protein kinases (MAPK), cyclic AMP dependent kinase, p21-activated kinases (PAK), PKB/AKT, protein kinase A (PKA), phosphatidylinositol-3-kinase (PI3K) and non-receptor associated protein kinases (e.g. JAK and SRC) (58, 96, 97).

NOX activators include cytokines (98), platelet derived growth factor (99), epidermal growth factor (50), TGF-β1 (100), mechanical forces such as pulsatile/oscillatory shear stress (101) cyclic stretch (102), hypoxia (52), and G protein coupled receptor agonists (55).

Protein-protein interactions among NOX and members of the thioredoxin family, and transient oscillations in intracellular concentration of various ions, may trigger the activation of NOX. Nuclear factor erythroid 2- related factor 2 (NrF2) is a negative regulator of NOX. Phosphorylation of p47-phox allows it to bind to a p40-phox-p67-phox complex (103)and facilitates the translocation of the trimer to the membrane where it binds to p22-phox thus assembling the active NOX complex (92).

It has recently been reported that NOX enzymes are present in extracellular vehicles (EVs) and microparticles (MPs) released from various cells, including endothelial cells (104, 105). EVs have been implicated in a number of pathological and physiological conditions such as cancer and atherosclerosis (106, 107). During septic shock, platelet derived exosomes may generate ROS through NOX-2, which forms ONOO- that induces endothelial apoptosis (106). NOX2 is present in circulating MPs from patients with hypercholesterolemia (108). However, as NOX2 activation requires the translocation of its cytosolic subunit p47 to the cytoplasmic membrane, it is important that p47 is also localized at the membrane surface or in proximity of the vesicle (85).

## Subclinical Atherosclerosis

**Figure 3:**
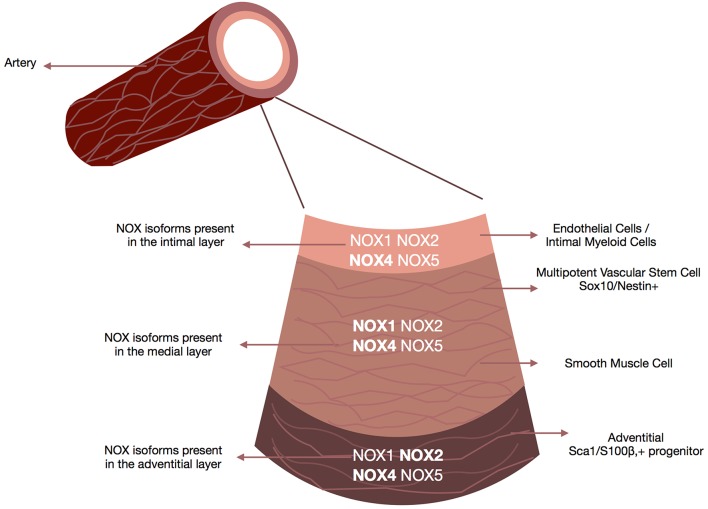
NOX enzymes present within the vascular walls. Schematic depicts the repertoire of NOX enzymes within all three layers of the vascular wall, the adventitia (i.e., fibroblasts, macrophages, and adventitial progenitor stem cells), the media (i.e., smooth muscle cells accounting for 90% of the vessel wall) and the intima (i.e., endothelial cells/smooth muscle cells). Although all isoforms are expressed at some levels within all three layers there are distinct NOX profiles associated with each layer. NOX4 is the predominant isoform in endothelial cells, NOX1 and NOX4 in smooth muscle cells, Nox4 in fibroblasts, and Nox2 and NOX4 in resident vascular stem cells.

The arterial wall is comprised of the intima (the innermost layer of endothelial cells with some intimal myeloid cells surrounded by a basal lamina), the media (consists mostly of smooth muscle cells and some resident stem cells supported by the extracellular matrix) and the adventitia (the outer most layer containing a variety of cell types including fibroblasts, myeloid cells, macrophages, adipocytes and pericytes) (109) (Figure 3).

Atherosclerosis is a chronic progressive inflammatory disease and the leading cause of death worldwide (6). Despite an extensive understanding of established/advanced lesion morphologies that lead to myocardial infarction or stroke due to thrombosis from acute plaque rupture or erosion, there still exists a superficial understanding of the initiation and progression of subclinical atherosclerosis. Central to this process is the development of non-atherosclerotic intimal lesions referred to as adaptive [AIT] or diffuse [DIT] intimal thickening, before the appearance of pathologic intimal thickening (PIT) leading to plaque formation in human vessels (7, 8, 110). Intimal thickening (AIT/DIT) leads to lipid deposition within the walls of the thickened artery and transpires before the build-up of foam cells which results in atheroma formation and restricted blood flow (1). Within the atheroma, there is also a significant accumulation of synthetic SMCs that are considered both protective when at the fibrous cap but also alternatively atherogenic if they become macrophage-like cells (6). Several studies have shown upregulation of NOX-based NAD(P)H oxidases during the progression of AIT following flow restriction due to carotid injury (111) and lipid diet (112). The importance of intimal thickening to subclinical atherosclerosis in advancing atherosclerosis has been clearly established in ApoE gene deficient mice fed on a western diet in combination with carotid artery ligation-induced injury (113, 114).

The origin of the SMC-like cells that contribute to DIT/AIT and make up the atheroma remains controversial. However, there are four proposed mechanisms based on recent lineage tracing analysis that include:

1. De-differentiation/reprogramming of a subset of medial vascular smooth muscle cells (15, 115)
2. Myogenic differentiation of a resident vascular stem cells (16, 116)
3. Myogenic differentiation of bone marrow-derived mesenchymal stem cells (17)
4. Endothelial-mesenchymal cell transition (EndMT) (18, 117)

## Vascular Role of ROS

In the vascular bed, the main ROS of interest generated by NOX are H_2_O_2_ and O_2_− (11). H_2_O_2_ in low concentration is a vital signaling molecule under physiological conditions whereas O_2_− is associated with oxidative stress leading to pro-inflammatory and oxidative processes (Figure 3).

Cardiovascular risk factors such as hypercholesterolemia (118), hypertension (100), diabetes mellitus (76), and smoking (119) all increase ROS generation and decrease endothelial NO production. There is compelling evidence to support the role of ROS in intimal thickening leading to the progression of atherosclerosis *in vivo* using rodent models (10, 71, 111). Adaptive vascular lesions preferentially form within regions of disturbed blood flow leading to enhanced ROS and pathologic intimal thickening (8), and numerous human studies have demonstrated several NOX proteins including gp91phox and NOX4 contribute to increased intracellular oxidative stress in a cell-specific manner and thus may be involved in the genesis and progression of human coronary atherosclerotic disease (118). *In vivo* studies on the specific role of NOX homologs in vascular lesions have significantly advanced our understanding of the role of superoxide and increased NOX expression in injury models in mice (120), while antioxidant treatment with tempol or N-acetyl-cysteine protects against injury-induced lesion formation (121).

### ROS in Vascular Endothelium

The vascular endothelium plays a critical role in vessel homeostasis by maintaining blood flow regulating blood flow, controlling macromolecule and fluid exchange with tissues and preventing leukocyte activation (122). NOX are in part localized to the plasma membrane, producing extracellular superoxide with a paracrine function but NOX 1, 2, and 4 are also localized in intracellular compartments with a perinuclear distribution (123). Sustained ROS levels contribute to endothelial dysfunction and activation of an inflammatory phenotype leading to the development of atherosclerosis (82). In vascular endothelial cells the main source of ROS is the electron leakage from the mitochondria (46).

Endothelial cell-dependent relaxation is primarily mediated by endothelial-derived hyperpolarizing factor (EDHF) and nitric oxide (NO), with H_2_O_2_ as the primary EDHF (39). NOX4 generates H_2_O_2_, which may react with NO and increase (124) or decrease (125) endothelial nitric oxide synthase (eNOS) expression and activity through a phosphoinositide 3-kinase-dependent and the inhibition of AP-1 activity, respectively. Flow, the preeminent stimulus for endothelial NO, also promotes the endothelial release of other factors that impact on vascular function, including activation of the lysosomal biogenesis transcription factor EB (TFEB) to decrease mTOR (mechanistic target of rapamycin) activity (80, 126). Indeed, ROS may induce autophagy by activating the major Ca^2+^ release channel on the lysosomal membrane through a TFEB pathway, facilitating the removal of damaged mitochondria and excess ROS (127). Moreover, athero-prone regions of the vasculature have enhanced TFEB levels linked with a reduction in H_2_O_2_, and superoxide (126).

Finally, proinflammatory lipids like lysophosphatidylcholines (LPC) are known to stimulate ROS formation in atherosclerosis, an effect that is attenuated by mitoTEMPO—a mitochondrial ROS scavenger. Therefore, an imbalance of redox-mediated signaling in endothelial cells may precipitate endothelial dysfunction that is a key event for the development of atherosclerosis (46).

NOX4 is also involved in endothelial progenitor stem cell proliferation, migration and cell survival after exposure to TNF-alpha (128). Superoxide (O_2_−) attenuates endothelial cell dependant relaxation, therefore SOD may play an important role to limit intracellular O_2_− concentration in endothelial cells by converting O_2_− to H_2_O_2_ (129).

### ROS in Vascular Smooth Muscle Cells

The primary NOX isoforms in SMCs are NOX1 and NOX4 (130). Their localization and enzymatic activity differs in that NOX1 is primarily found in the plasma membrane, caveoli and endosomes, whereas NOX4 localizes to focal adhesions, the endoplasmic reticulum, and mitochondria. NOX1 interacts with multiple regulatory proteins to drive inducible O_2_−, while NOX4 is constitutively active and primarily generates H_2_O_2_ (131). NOX1 and NOX4 have highly specialized roles within smooth muscle cells (SMCs). Evidence suggests that NOX4 is involved in maintenance of SMC quiescence (132) while NOX1 has a role in modulating SMC function (33, 133). Most data to date has come from global transgenic murine studies that reveal the overall effect of NOX1 and NOX4 in normal and disease development (86). Ectopic expression of NOX1 in vascular smooth muscle promotes the production of ROS in response to ANG II and causes eNOS uncoupling and a decrease in nitric oxide bioavailability, resulting in impaired vasorelaxation (134). More recent studies have focused on SMC-specific transgenics that allow definition of the specific contribution of SMC NOX proteins to SMC function and phenotypic state, and to vascular disease development (86, 135). Using NoxO1 or p47phox gene deleted animals, ROS production stimulated by NoxO1 and p47phox limited endothelium-dependent relaxation and maintained blood pressure. However, NoxO1 and p47phox cannot substitute each other despite their similar effects on vascular function. Deletion of NoxO1 induced an anti-inflammatory phenotype, whereas p47phox deletion rather elicited a hyper-inflammatory response (136).

Activation of NOX1 contributes to matrix degradation, and the migration and proliferation of SMC (137). Protein kinase C-beta1-mediated phosphorylation of NOX1 is necessary for its interaction with the NOXA1 subunit and generation of superoxide (138). A peptide inhibitor of this process prevents SMC migration (139). Induction of SMC proliferation and hypertrophy leads to downregulation of NOX4 and upregulation of NOX1 expression, respectively. NOX4 downregulation leads to senescence of human vascular smooth muscle cells (140).

PDGF-BB-induced increases in NOX1 expression and H_2_O_2_ production promotes activation of c-Jun N-terminal kinase (JNK), cyclin D and extracellular signal-regulated kinase (ERK)1/2 signaling to enhance SMC migration and proliferation, respectively (141). On the other hand, Ang II-induced SMC hypertrophy is regulated by NOX1 activation of Ras, p38 mitogen kinase activated protein kinase (MAPK)/protein kinase B (Akt), and epidermal growth factor (EGF) receptor pathways (142). NOX1 mRNA expression is enhanced in phenotypic de-differentiated SMC (112). NOX1 deficient mice exhibit decreased proliferation and migration in response to PDGF-BB, whereas ectopic expression of NOX1 has the opposite effects (86).

In further agreement, ectopic SMC expression of human NOX1 facilitated enhanced Ang II-induced vascular O_2_− production, hypertension and vessel wall hypertrophy (143). Contradictory data from global NOX1 knockdown suggest that NOX1 deficiency may be protective after femoral wire-induced injury by attenuating neointima formation and cell proliferation (86). Indeed, vascular NOX1 levels are upregulated in carotid arteries following balloon injury. Utilizing a combination of genetic mouse models and cell culture studies, strong evidence has recently emerged that the NOX1 coactivator protein, NoxA1 also critically regulates SMC migration and phenotypic modulation in stenotic and atherosclerotic vascular remodeling (112, 144, 145).

In contrast to NOX1, NOX4 function is associated with SMC contractile proteins *in vitro* and maintenance of SMC in a quiescent contractile state (132). NOX4, as the primary isoform in SMCs is responsible for the baseline levels of ROS in maintaining the identity of differentiated SMCs (146). Indeed, NOX4 knockdown results in a decrease in SMC differentiation marker expression [smooth muscle myosin heavy chain 11 (SM-MHC), smooth muscle alpha actin (ACTA-2) and calponin 1 (CNN1)], while NOX4 overexpression increases their expression. NOX4 specifically may be required for maintenance of the contractile-type stress fibers in SMCs. TGF-β1 stimulates SMCs differentiation and specifically induces H_2_O_2_ generated by NOX4 via the SMAD signaling pathway (132). NOX4 mediates TGF-β1 induced SMC proliferation but not that by PDGF-BB or interferon gamma. NOX4 knockdown also results in decreased levels of serum response factor (SRF) required for CArG box dependent expression of SMC contractile proteins. NOX4 and CNN1 are both expressed within the neointima following balloon injury while ectopic SMC expression of a NOX4 dominant negative mutant reversed neointima formation following injury, in part by suppressing epoxide hydrolase 2 which inhibits SMC proliferation, migration and inflammation (147).

While not expressed in rodent vessels, NOX5 is present in human and porcine cells and is preferentially expressed in human coronary arteries and atherosclerotic lesions (43, 148). Putative regulators of NOX5 include interferon-gamma (IFNγ) which increases NOX5 production (43) while NOX5 knockdown impairs PDGF-BB mediated proliferation and ROS production (149)

Collectively, coordination of NOX1 and NOX4-dependent signaling facilitates de-differentiation and subsequent migration and proliferation of SMCs. Strategies such as those to inhibit NOX1 phosphorylation or NOX4 silencing may mitigate the development of cardiovascular disease.

### ROS in Adventitial Cells

The adventitia used to be considered as an inert connective layer comprised of adventitial fibroblasts wrapped around the medial layer. However, accumulated data now suggests that the adventitia is a major site of immune and inflammatory cell trafficking that is facilitated by the vasa vasorum to maintain the medial layer and provide an important gateway for macrophage and leukocyte migration into the intima (150). It is also an important stem/progenitor cell niche ready to respond to arterial injury and thus acts as an essential regulator of vascular wall structure by contributing to the reorganization of the extracellular matrix (151). Adventitial cells express NOX1, NOX2, and NOX4 isoforms (152). NOX2, p22phox, p47phox, and p67phox are abundantly expressed in aortic vascular adventitia and in cultured adventitial fibroblasts *in vitro* (153). In contrast, NOX4 is weakly expressed in adventitial fibroblasts of human coronary arteries (118). NOX can be activated in adventitial cells by the similar vascular different stimuli including cytokines, hormones, metabolic factors and mechanical injury to stimulate the release of ROS (152). NOX1 and 4 are associated with hypoxic challenge in the adventitia (95). The primary role of NOX within the adventitia may be superoxide production and increased expression of adhesion molecules leading to chemotactic movement of leukocytes and their increased penetration into the vessel wall (142). Human coronary artery adventitial fibroblasts express NOX2 and NOX4 that produce superoxide in response to angiotensin II (Ang II) (118). This superoxide can be converted to H_2_O_2_ by extracellular SOD thus raising the possibility that H_2_O_2_ derived directly from NOX4 in the adventitial layer can also act as a paracrine mediator.

### ROS in Intimal Myeloid Cells

Myeloid cells are present in the intima of the large arteries like the aorta, where vascular lesions and atherosclerosis plaques develop (154). NOX are primary sources of ROS in macrophages where a tumor necrosis factor–like weak inducer of apoptosis (TWEAK) fibroblast growth factor–inducible 14 (Fn14) TWEAK/Fn14 axis regulates NOX2-dependent ROS production (83). While it remains unclear whether endogenous NOX in macrophage has a direct impact on the progress of atherosclerosis, many studies have revealed a significant role of NOX-derived ROS in regulation of monocyte differentiation and macrophage functions (155). Recent evidence also indicates that human monocytes and macrophages express functionally active NOX5 (43) and that a NOX5-p22phox complex drives macrophage-dendritic differentiation (156).

Mitochondrial ROS is also an important source of ROS in macrophages and promotes MCP-1 production to promote monocyte infiltration and lesion inflammation (157). Another potential source of ROS in macrophages is the XO. XO inhibitors inhibit macrophage ROS formation, inflammatory cytokine release, and atherosclerosis (158). XO breaks down hypoxanthine and xanthine to uric acid and produces ROS, both of which may affect the function of macrophages. However, XO-dependent generation of ROS, rather than uric acid, mediates inflammatory cytokine production (159). Irrespective of origin, ROS can also significantly affect macrophage function whereby heme scavengers inhibit heme-mediated ROS production and ROS-mediated oxidative damage (160).

## Pathological Role of ROS

**Figure 4:**
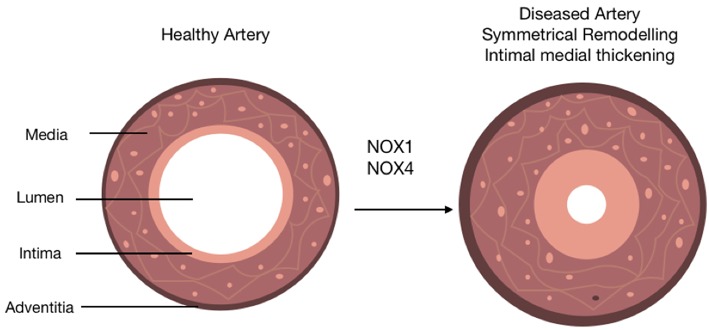
The role of NOX isoforms in vascular disease progression. Schematic represents the role of NOX 1/4 enzymes in the progression of arteriosclerosis. A healthy artery is depicted with three distinct layers, the outermost layer; the adventitia, the middle layer; the media and the innermost layer; the intima. The activity of NOX 1/4 enzymes and subsequent production of ROS leads to the progression of arteriosclerosis. This is characterized by the accumulation of neointimal SMC-like cells within the medial and intimal layers (intimal medial thickening), an induction of adventitial fibrosis represented by a slight increase out the adventitia, and a distinctive narrowing of the lumen subsequently resulting in restricted blood flow.

Oxidative stress in arteriosclerosis results primarily from the activity of NOX enzymes (9) (Figure 4). However, their specific role in SMCs during the progression of subclinical arteriosclerosis remains unresolved. While global NOX1 deficient mice develop less neointimal thickening after wire-induced injury consistent with subdued SMC proliferation and migration rates *in vitro* and enhanced NOX1 expression in neointimal SMCs *in vivo*, SMC-specific ectopic expression of NOX1 failed to increase DIT/AIT (86). This enhanced NOX1 expression was associated with ERK1/2 (extracellular signal-regulated kinases 1/2) activation and enhanced MMP-9 (matrix metallopeptidase 9) (33). Gene expression network analysis of human arteriosclerotic vessels suggests the network hub gene glutathione peroxidase-1 (GPX1) is the most significantly downregulated following pathologic intimal thickening (161). Decreased GPX1 expression in atherosclerotic mice led to reductive stress via a time-dependent increase in glutathione suggesting that GPX1-dependent alterations in oxido-reductive stress promote vascular remodeling.

Endothelial NOX4 plays a critical role in the control of atherosclerosis where ROS is athero-protective via NOX4-dependent inhibition of inflammation and vascular remodeling (162). Ectopic expression of endothelial NOX4 in ApoE deficient mice reduced lesion formation, increased Treg numbers and decreased levels of effector T cells and chemokines (162). However, downregulation of NOX4 in human aortic ECs increased the expression of profibrotic CTGF (connective tissue growth factor), while decreasing endothelial H_2_O_2_ and reducing the levels of p-SMAD3 (phosphorylated mothers against decapentaplegic homolog 3) (40). NOX4 knockdown *in vivo* also leads to increased fibrillar collagens I and III in plaques, which is associated with elevated transforming growth factor-β expression and p-SMAD3 levels in diabetic lesions (135). The response of endothelial cells to endoplasmic reticulum (ER) stress during the progression of arteriosclerosis is governed by NOX4 and H_2_O_2_ (163). ER stress increases H_2_O_2_ in ER in a NOX4-dependent manner leading to oxidation of Ca^2+^-ATPase, elevated cytosolic calcium and RasGRF (Ras-specific guanine nucleotide releasing factor) activation. NOX generated ROS also impacts on XBP1 splicing (X-box-binding protein 1), a key protein that promotes EC apoptosis and atherosclerosis formation (164).

**Figure 5:**
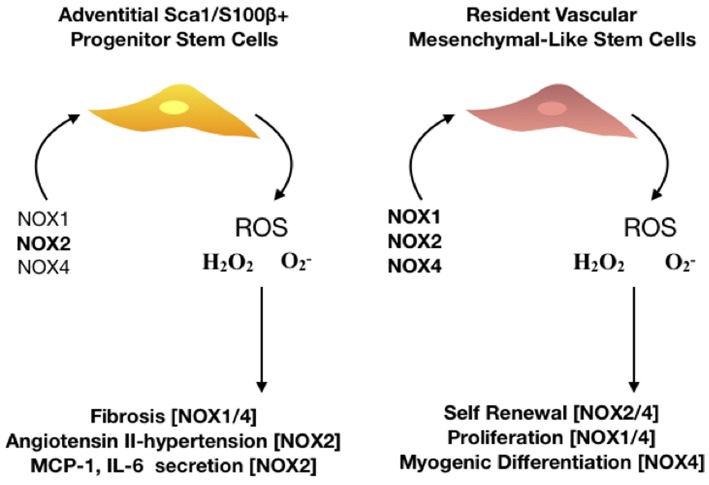
The role of NOX isoforms in vascular stem cell populations. Schematic represents the effect of NOX 1,2, and 4 enzymes on stem cell activity within the vasculature. There are two major resident stem cell populations that are effected by NOX enzymes, adventitial Sca1/S100β+ and resident vascular mesenchymal-like stem cells. NOX2 is predominantly associated with hypertensive vessels and promotes the secretion of monocyte chemoattractant protein (MCP-1) and interleukin 6 (IL-6) whilst NOX 1/4 are associated with hypoxic challenge and fibrosis in adventitial progenitor cells. Proliferation, self-renewal and differentiation of resident vascular stem cells is driven by NOX 1,2, and 4 as the production of ROS- mediates P13K/AKT dependent signaling whilst orchestrating a redox-mediated regulatory mechanisms of stem cell function of vascular repair.

The presence of NOX4 in adventitial fibroblasts and the adventitial location of ROS production following AIT/DIT in murine models of vascular remodeling highlights their fundamental importance to vascular pathology and regeneration (165) (Figure 5). The functional significance of NOX4 in adventitial fibroblasts has led investigators to suggest an “outside in” process of vascular remodeling. p22phox protein and ROS production both increase within the adventitial layer of injured carotid arteries (111) while NOX4 overexpression stimulates migration and proliferation, as well as matrix gene expression, of adventitial fibroblasts (166). NOX4 also mediates TGF-β1 activation of fibroblasts, which promotes differentiation into a profibrotic myofibroblast phenotype and matrix production (167). Small molecule inhibitors of NOX4 reduce adventitial ROS generation and subsequent vascular remodeling (89). This “outside in” process is further supported by recent lineage tracing analysis using Gli-Cre-LoxP transgenic mice supporting a role for adventitial cells in contributing to DIT/AIT through hedgehog signaling pathways (151, 168) Notably, interaction of NOX4 with hedgehog has recently been demonstrated in gastric cancer cells (169). Overexpression of the hedgehog target gene, Gli1, inhibited the anti-mitogenic effect of NOX4 knockdown while concomitant overexpression of NOX4 increased Gli1 expression, an effect reversed by Gli1 depletion. Further, ROS generated by NOX4 was required for GLI1 expression, as shown by use of the ROS inhibitor, diphenylene iodonium (DPI).

In arteriosclerosis, innate and adaptive immune cells also enter the vasculature and participate in the pathology of AIT and PIT by releasing several mediators including ROS that cause vascular damage (170). Upon exposure to hypercholesterolemia, these cells get loaded with oxidized LDL and acquire foam cell morphology prior to the recruitment of monocytes that differentiate into macrophage foam cells. Atherosclerotic patient-derived monocytes/macrophages have exaggerated IL-6 and IL-1β levels, which was highly dependent on mitochondrial ROS but not NOX2 (171). Moreover, 8-oxoguanine glycosylase, a major DNA glycosylase responsible for removing mitochondrial oxidative stress–induced DNA damage, plays a protective role in atherosclerosis by preventing excessive inflammasome activation in macrophages, further supporting the critical role of macrophage mitochondrial oxidative stress in promoting atherosclerosis. While macrophages produce ROS through XO, there is still a lack of solid evidence demonstrating the role of macrophage XO in atherosclerosis (172).

## ROS and Resident Vascular Stem Cells

It is widely accepted that the regulation of stem cell self-renewal and differentiation is crucial for tissue homeostasis, and in particular, vascular remodeling and fibrosis (173). Indeed several recent studies have highlighted the critical importance of the cellular oxidation-reduction (redox) state in modulating the balance between stem cell self-renewal and differentiation (41, 174). As a result, the study of ROS regulation in regenerative medicine has rapidly evolved to define the putative roles of oxidative stress in dictating the fate of multi-potent and pluripotent stem cells.

In adult vessels, resident vascular stem cells (rVSCs) are present in all three layers and are important in maintaining vessel homeostasis (16, 151, 175) These progenitor cells express various stem cell markers including Sca-1, cKit, CD34 and Flk1, S100β, Sox10, Sox17, and Nestin, are multi-potent and can differentiate into lineages of mesoderm and neuroectoderm origins including SMCs, osteoblasts, adipocytes and chrondrocytes (173). The influence of rVSC behavior on the development of subclinical atherosclerosis may be critical. Hence, a greater understanding of the regulatory mechanisms that control stem/progenitor cell expansion, migration, and differentiation is essential for targeted therapies. Accumulating evidence suggests that rVSCs are mobilized by local signal molecules in their microenvironment. Importantly, rVSCs are normally quiescent but can be activated in response to injury to participate variously in endothelial regeneration, adventitial fibrosis and/or neointimal SMC-like cell accumulation that drives subclinical arteriosclerosis and neointima formation (173). Cell fate mapping studies using transgenic mouse models have greatly extended our understanding of the fate of these cells and that of their progeny during pathologic vessel remodeling, and how they might be influenced by the redox state (71). While little is known about ROS control of resident vSCs, in general, these progenitors adopt a mesenchymal stem-like phenotype before they undergo myogenic differentiation to SMC-like cells and accumulate with the neointima during subclinical atherosclerosis (116). Vascular mesenchymal-like stem cells (MSCs) are multipotent stem cells that are defined by three main characteristics: plastic adherence, ability to naturally differentiate into a diverse set of tissues within the mesoderm lineage, and of self-renewal (173).

It is clear that NOX–derived ROS is a major regulator of MSC cell fate (41). There are conflicting reports on the antioxidant levels at baseline for MSCs and their subsequent resistance to oxidative stress (176, 177). This may be due to differences in the timing of MSC isolation, cultivation and exposure to oxidative insult as cellular senescence and cell age increase oxidative stress. Nevertheless, NOX1 and NOX4 derived ROS is hypothesized to be a redox messenger for rVSC derived MSC proliferation and differentiation (178). In general, low levels of ROS are associated with MSC maintenance and expansion in pluripotent embryonic stem cells (ESCs) and multipotent adult stem cells, whereas increased levels are associated with stem cell differentiation (174, 179). Pharmacological or genetic approaches to alter stem cell metabolism have been shown to directly influence stem cell activity since ROS generation in both embryonic and adult stem cells is mainly dependant on glycolysis (180). There is a large difference in energy metabolism and cellular redox status between pluripotent stem cells and terminally differentiated cells. As low levels of ROS are required in stem cells to maintain quiescence and self-renewal, it is likely that rVSCs and their MSC progenitors reside in a specialized microenvironment that is low in O_2_ (41).

Physiological low levels of ROS, either from exogenous H_2_O_2_ or hypoxia, play an important role in the regulation of MSC cell fate decision through activation of NOX-1 and NOX-4 (181, 182). In particular, ROS regulates cell expansion by (i) activation of miR-210 that triggers ERK1/2 and AKT activation in MSCs (ii) secretion of chemokines (e.g., CCL-2, CCL-4) through the activation of p38-mitogen-activated protein kinases (MAPK) pathway (183) and (ii) release vascular endothelial growth factor (VEGF) to promote angiogenesis (184). In contrast, high endogenous levels of ROS not only promote oxidative stress to disrupt adhesion through the down-regulation of key focal adhesion molecules including focal adhesion kinase (FAK), Src, and integrin expression (185), but also DNA damage (186) and a reduction in telomere length leading to MSC senescence (187). Oxidative stress also causes cell cycle arrest by inhibiting phosphorylated retinoblastoma (pRB) expression via a p38 MAPK/P16 pathway (188), disrupts mitochondrial cardiolipin-cytochrome c complexes and induces BAX-BAK dimerization to drive overall apoptosis and cell death (189). Finally, ROS may alter stem cell fate by influencing the epigenetic landscape via DNA methylation, post-translational histone modifications, ATP-dependent alterations to chromatin and non-coding RNA transcripts (69).

### Myogenic Differentiation of Stem Cells- the Role of ROS

There is a direct correlation between NOX-derived ROS and modulation of multiple stem fates. Differentiation of mesenchymal stem cells is stimulated by differentiation factors such as TGF-β (190), epidermal growth factors (EGF) (191), wingless type MMTV integration site (wnt) proteins (192), fibroblast growth factor (FGF) (193). Many redox sensor proteins play a key role in altering stem cell fate, such as transcription factors NF-κB (194), forkhead box O (FOXO) (195), nuclear factor erythroid 2 (NRF2) (103) and the p53 (TRP53) tumor suppressor (196). Differentiation signals induce NOX4 in stem cells suggesting intracellular H_2_O_2_ may act as a non-specific intracellular differentiation signal and influence other activated pathways in promoting specific lineages (197).

MSCs undergo myogenic differentiation to SMC like-cells upon stimulation with TGF-β1, mechanical stress and sphingosylphosphorylcholine (SPC), and with co-cultivation with vascular endothelial cells (198). Little is known about how ROS contributes to myogenesis in rVSC but SPC promotes SMC differentiation in human MSCs, which is dependent on ROS activation of the DJ-1 pathway (199). ROS can also induce myogenic differentiation during the early stages in various stem cells when NOX4 is activated by TGF-β1 and/or PDGF-BB to generate ROS (H_2_O_2_ and O_2_−). NOX4-derived H_2_O_2_ up-regulates serum response factor (SRF) gene transcription and protein translation, which when phosphorylated binds CArG elements within the promoter-enhancer region of SMC-specific genes to regulate myogenic differentiation (132). NOX4-derived O_2_− also activates MAPK which increases SRF-mediated gene transcription activation to further drive differentiation (139, 200). At the later stage of myogenic differentiation, NOX4 is recruited to SMC myofilaments to maintain cells in a differentiated state (197). NOX4 also drives myogenic differentiation from mouse embryonic stem cells (ESCs) *in-vitro*, mediated by NRF-2 (200). Anti-oxidants such as selenium may also dictate stem cell differentiation, as they influence the cellular redox profile (201).

### Endothelial to Mesenchymal Transition (EndMT)

Endothelial to mesenchymal transition (EndMT) is a process whereby an endothelial cell undergoes a series of molecular events that lead to a change in phenotype toward a mesenchymal cell (e.g., myofibroblast, smooth muscle cell) (202). EndMT plays a fundamental role during development, and recent evidence suggests that EndMT may be involved in cardiovascular diseases (CVDs), including atherosclerosis, pulmonary hypertension, valvular disease, and fibroelastosis. In particular, EndMT has been implicated in the progression of subclinical atherosclerosis as “transitioning” cells and is readily detected in human plaques (117). Oxidative stress is known to be involved in EndMT and subsequent vascular damage through TGF-β (203). Brain Arnt-like protein-1 (BMAL1) suppresses ROS production and a positive relationship exists between loss of BMAL1 expression and EndMT in atherosclerotic plaque vulnerability in human carotid plaques. *In-vitro*, BMAL1 inhibits oxidized low-density lipoprotein-induced intracellular ROS accumulation and subsequent EndMT in human aortic endothelial cells (204–206). Endothelial-specific NOX2 overexpression in transgenic mice enhances EndMT and has pronounced pro-fibrotic effects in the heart (207). Moreover, exposure of endothelial cells to H_2_O_2_ inhibits endothelial specific lineage genes and promotes EndMT due to TGF-β (208). Similarly, hypoxia promotes EndMT of human endothelial cells (117) by upregulating unregulated EndMT genes, SNAIL1 and SNAIL2 (209).

## Antioxidants Effect on Atherosclerotic Disease

Extensive Cochrane meta-analysis of clinical studies suggests no significant benefit of anti-oxidants in CVD (210). Despite this, higher dietary intake and/or blood concentrations of vitamin C, carotenoids, and α-tocopherol (as markers of fruit and vegetable intake) were all associated with reduced risk of cardiovascular disease independent of anti-oxidant effects (211). Nevertheless, patient cohorts at high risk of cardiovascular disease exhibit a low plasma concentration of anti-oxidants such as β-carotene, α-tocopherol, and ascorbic acid (212). Epidemiological studies confirm an inverse relationship between plasma concentration of anti-oxidants and degree of atherosclerotic disease (213). EUK-207, an anti-oxidant therapy reduces endothelial P-selectin, von Willebrand factor A1-domain and platelet adhesion in mouse models of atherosclerosis concomitant with reduced plaque area and macrophage content. ROS scavenging may also affect stem cell differentiation, as complete scavenging with a flavoprotein inhibitor NAC completely inhibited human embryonic stem cell derived CD34+ differentiation (214). In general, anti-oxidants are beneficial to stem cell activities by [i] mitigating oxidative stress through neutralization of free radicals and increasing the expression of antioxidant enzymes and [ii] influencing the differentiation fate of precursor stem cells

Patient cohorts with high plasma concentrations of reductants including cryptoxanthin, lycopene and α-carotene have lower intimal thickening compared to subjects with a low concentration of these anti-oxidants (215), and an inverse correlation exits for α-carotene and β-carotene and subclinical atherosclerosis (216). Lycopene, which has the highest reducing capacity among the carotenoids (650 mV), significantly decreased plaques in transgenic mice while improving endothelial function (217). Moreover, there is an inverse correlation between the plasma concentration of the anti-oxidant Vitamin E and the development of cardiovascular diseases (210, 218). Another anti-oxidant, selenium (Se), is incorporated into proteins known as selenoproteins to reduce H_2_O_2_ and lipid/phospholipid hydro-peroxidases by Se-dependent glutathione peroxidases (GPXs), while low levels of GPx-1 (the primary selenoprotein in mammals) activity are associated with atherosclerosis and severity of disease (219). Finally, despite limited bioavailability and rapid degradation, dietary anthocyanins are antioxidants with potentially significant cardiovascular benefits (220).

## Conclusions

Subclinical atherosclerosis is characterized by intimal thickening due to the accumulation of neointimal SMC-like cells derived from a heterogeneous population of parent cells including differentiated SMCs, resident vascular stem cells, bone-marrow derived MSCs and EndMT. The heterogeneity reflects the variable phenotypes and functions of these cells depending on the severity of the injury to the vessel wall. Indeed, many investigators are speculating that these phenotypes may represent the various different stages of resident stem cell mediated differentiation. Redox/ROS signaling through the activity of NOX and or mtROS controls the maintenance of these phenotypes and their contribution to intimal thickening and subclinical atherosclerosis.

While global and cell-specific knockdown studies have presented compelling evidence for the role of NOX isoforms in controlling cell fate during intimal thickening, similar studies that address the functional consequences of NOX knockdown on specific stem cell populations and intimal myeloid cells are required. Moreover, elucidation of the mechanisms dictating the migration, proliferation, and myogenic differentiation of resident vSCs vascular stem cells through NOX-dependent pathways will provide vital information for the development of more targeted therapies for treating subclinical atherosclerosis.

## Author Contributions

DB and MK wrote the manuscript. DB and PC prepared the figures. MK prepared the tables. IM, PC, and ER reviewed and edited the manuscript.

### Conflict of Interest Statement

The authors declare that the research was conducted in the absence of any commercial or financial relationships that could be construed as a potential conflict of interest.
